# Machine Learning and Statistical Shape Modelling Methodologies to Assess Vascular Morphology before and after Aortic Valve Replacement

**DOI:** 10.3390/jcm13154577

**Published:** 2024-08-05

**Authors:** Yousef Aljassam, Froso Sophocleous, Jan L. Bruse, Vico Schot, Massimo Caputo, Giovanni Biglino

**Affiliations:** 1 Department of Translational Health Sciences, Bristol Medical School, University of Bristol, Bristol BS2 8HW, UK; ce21129@bristol.ac.uk (Y.A.); fs16815@bristol.ac.uk (F.S.); v.schot@bristol.ac.uk (V.S.); m.caputo@bristol.ac.uk (M.C.); 2 Fundación Vicomtech, Basque Research and Technology Alliance BRTA, Mikeletegi 57, 20009 Donostia-San Sebastián, Spain; jbruse@vicomtech.org; 3 Bristol Heart Institute, University Hospitals Bristol and Weston NHS Foundation Trust, Bristol BS2 8HW, UK

**Keywords:** statistical shape modelling, hierarchical clustering, aortic valve replacement, aortic morphology, medical imaging

## Abstract

**Introduction**: Statistical shape modelling (SSM) is used to analyse morphology, discover qualitatively and quantitatively unique shape features within a population, and generate mean shapes and shape modes that show morphological variability. Hierarchical agglomerative clustering is a machine learning analysis used to identify subgroups within a given population in relation to shape features. We tested the application of both methods in the clinically relevant scenario of patients undergoing aortic valve repair (AVR). Every year, around 5000 patients undergo surgical AVR in the UK. **Aims**: Evaluate aortic morphology and identify subgroups amongst patients who had undergone AVR, including Ozaki, Ross, and valve-sparing procedures using SSM and unsupervised hierarchical clustering analysis. This methodological framework can evaluate both pre- and post-surgical variability across subgroups undergoing different surgeries. **Methods**: Pre- (*n* = 47) and post- (*n* = 35) operative three-dimensional (3D) aortic models were reconstructed from computed tomography (CT) and cardiac magnetic resonance (CMR) images. Computational analyses for SSM and hierarchical clustering were run separately for the two subgroups, assessing (a) ascending aorta only and (b) the whole aorta. This allows for exploring possible variations in morphological classification related to the input shape. **Results**: Most patients in the Ross procedure subgroup exhibited differences in aortic morphology from other subgroups, including an elongated ascending and wide aortic arch pre-operatively, and an elongated ascending aorta with a slightly enlarged sinus post-operatively. In hierarchical clustering, the Ross aortas also appeared to cluster together compared to the other surgical procedures, both pre-operatively and post-operatively. There were significant differences between clusters in terms of clustering distance in the pre-operative analyses (*p* = 0.003 for ascending aortas, *p* = 0.016 for whole aortas). There were no significant differences between the clusters in post-operative analyses (*p* = 0.47 for ascending, *p* = 0.19 for whole aorta). **Conclusions**: We demonstrated the feasibility of evaluating aortic morphology before and after different aortic valve surgeries using SSM and hierarchical clustering. This framework could be used to further explore shape features associated with surgical decision-making pre-operatively and, importantly, to identify subgroups whose morphology is associated with poorer clinical outcomes post-operatively. Statistical shape modelling (SSM) and unsupervised hierarchical clustering are two statistical methods that can be used to assess morphology, show morphological variations, with the latter being able to identify subgroups within a population. These methods have been applied to the population of aortic valve replacement (AVR) patients since there are different surgical procedures (traditional AVR, Ozaki, Ross, and valve-sparing). The aim is to evaluate aortic morphology and identify subgroups within this population before and after surgery. Computed tomography and cardiac magnetic resonance images were reconstructed into 3D models of the ascending aorta and whole aorta, which were then input into SSM and hierarchical clustering. The results show that the Ross aortic morphology is quite different from the other aortas. The clustering did not classify the aortas based on the surgical procedures; however, most of the Ross group did cluster together, indicating low variability within this surgical group.

## 1. Introduction

Three-dimensional (3D) statistical shape modelling (SSM) is used to analyse morphology both qualitatively and quantitatively based on 3D imaging-derived reconstructions, providing insights as to features that otherwise cannot be adequately captured by analysing conventional 2D imaging [1]. Essentially, SSM allows parameterizing 3D shapes in one common mathematical framework so that it can be used to create an average shape of a shape population or discover unique 3D features of shape variability within a population by applying mathematical techniques. SSM has been used in different fields, such as medical imaging, anatomy, and engineering [1,2]. By applying principal component analysis (PCA) within a SSM framework, the dimensionality of a complex dataset can be reduced in order to facilitate data interpretability and analysis [3]. PCA is used to produce shape modes, each one relating to a certain percentage of the overall shape variability within the population and thus often representing a specific shape deformation, e.g., dilatation, curvature, or size [4]. This allows us to observe overall shape deformations within a population, assess relationships between morphology and pathology, or assess how a surgical procedure of interest may result in more or less marked anatomical changes [5].

A complementary statistical method to SSM, hierarchical agglomerative clustering is an unsupervised machine learning method used to identify subgroups within a given population in relation to shape features [6]. In general, this method has been found to be very useful to discover unique patterns in different fields, including genomics and taxonomy; for example, successfully identifying different subsets of cancers based on gene expression [7,8]. Cluster analysis measures similarities within the sample data and segments a sample of shapes into different groups, or clusters. Subjects’ similarity or dissimilarity is determined by calculating distances between subject feature vectors (in our case, shape vectors) using distance metrics, and clusters are formed by applying linkage functions. Each cluster has a set of similar shapes, and the shapes in other clusters are considered dissimilar [9]. This is visually represented by a dendrogram, a tree-like diagram that illustrates branches of clusters [10]. Agglomerative clustering is performed, considering each object as its own cluster and then calculating pairwise similarity, whereby the two most similar objects/clusters are grouped together, forming a single bigger cluster [11]. This process is repeated iteratively for every cluster, resulting in one large cluster at the top of the hierarchical tree, including all the objects. The identification of subgroups with this approach has the potential to improve diagnostic and treatment strategies [12]. This method is unsupervised, which is an appealing feature as the analysis does not necessitate knowledge about the population in question and does not require the indication of the number of clusters [9]. 

Here, we sought to test the application of both SSM and hierarchical agglomerative clustering in a clinically relevant scenario, i.e., aortic valve repair. Aortic valve repair for the treatment of aortic stenosis (AS) and regurgitation (AR) has improved greatly over the decades. Despite recent advances and good clinical outcomes [13], there is not one single strategy to repair the aortic valve. There are in fact numerous surgical procedures that have been developed and are used for aortic valve repair, including aortic valve replacement (AVR), i.e., replacing the diseased valve with either a mechanical valve or a bioprosthesis, as well as the Ozaki, Ross, and valve-sparing (VS) procedures [14,15]. While these all yield good clinical outcomes, they also present limitations, including some related to the material used for valve (re)construction and to the surgical technique itself. Other important factors include the shape and size of the patch used in repair [16], with prosthesis-patient mismatch occurring in up to 70% of procedures [17]. This may have serious clinical implications, as the mortality rate can almost double in severe mismatch cases [18]. Such a mismatch usually occurs when the prosthetic valve (the effective orifice area) is considered too small for the patient [19]. This mismatch can also impact the rate of degeneration of the prosthetic valve, negatively affecting cardiac function [17]. On top of known clinical differences, there may also be differences related to the morphology of the aortic root, ascending aorta, and whole aorta in terms of the suitability of a patient for a type of repair, and/or possible deleterious effects post-surgery related to the morphology of the aorta (e.g., dilation, tortuosity, curvature) [20]. Novel computational tools to study in depth the morphology of the aorta are therefore appealing and relevant. 

In this study, we aimed to evaluate aortic morphology and identify subgroups in a population of patients who had undergone AVR, Ozaki, Ross, and valve-sparing procedures using both SSM and unsupervised hierarchical clustering analysis in order to perform an in-depth analysis of 3D shape features within this population, which may impact future AVR intervention strategies.

## 2. Methods

The study comprised a pre-operative analysis and a post-operative analysis. The rationale for the pre-operative analysis relates to exploring whether the choice of surgery is associated with the morphology of the aorta. The rationale for the post-operative analysis relates to exploring the extent to which different surgeries affect aortic morphology. In both cases, analyses were performed on the isolated ascending aorta (referred to as the “ascending aorta”) and on a larger portion of the aorta that includes the arch and descending tract up to the level of the diaphragm (referred to as the “whole aorta”). From a methodological standpoint, running analyses including these two different input shapes is of interest, as it may reveal the impact of input shapes (including subtle changes having more/less weight in either scenario) on cluster allocation. As detailed below, the analyses included performing SSM analysis in addition to plotting a scatterplot of Mode 1 vs. Mode 2 and performing a hierarchical cluster analysis to classify aortas and detect variability across the surgical groups.

### 2.1. Patient Population

Patients who underwent aortic valve surgery (AVR, Ozaki, Ross, and VS) at the Bristol Heart Institute (2015–2022), with a computed tomography (CT) scan (Siemens (Siemens Healthineers, Forcheim, Germany); 1 mm reconstruction every 0.7 mm or Canon Aquilion (Canon Medical Systems Corporation, Ōtawara, Tochigi, Japan) 1; 0.5 mm reconstruction every 0.5 mm, 80/120 kV, 20 mg iodinated contrast per kg for 20 s duration) or cardiac magnetic resonance (CMR) scan (1.5T Siemens, temporal resolution 30 ms, matrix 156 × 256 with pixel size of 1 mm and slice thickness of 5 mm), were retrospectively included in the study. Patients with appropriate images were included, defined as: including the aortic arch and the descending aorta up to the diaphragm level, having adequate contrast in CT images, and having appropriate sequences for segmentation (non-cine sequences, sequences with a good number of slices > 60 slices) in CMR images. For the purpose of this feasibility study, patients with other pathologies, such as aortic coarctation, or who had previous interventions, such as stenting, were included regardless of their valve morphology (unicuspid, bicuspid, tricuspid). 

Ethical approval was waived by the local R&I department in light of the retrospective nature of the study and all imaging data were anonymized.

### 2.2. Segmentation and 3D Reconstruction

Medical images (DICOM) were segmented using commercial software (Mimics v25, Materialise, Leuven, Belgium). All reconstructions were performed by one person. The segmentation protocol included global thresholding and the split mask tool to only include the desired anatomy, which was then exported to 3-matic (Materialise) for smoothing and remeshing. The models were consistently cut below the aortic root (aortic valve annulus level) and at the diaphragm level. The brachiocephalic branches were trimmed as close as possible to the arch, isolating the morphology of the aorta. The anatomical landmark for cutting the ascending aortic models was the innominate artery (i.e., cutting with a plane perpendicular to the centreline of the aorta, as close as possible to the innominate). The difference between CT and CMR images as input data may yield very small resolution differences, which are not a concern for a large anatomy such as the aorta. In addition, all models were remeshed with a mesh size of 2 mm, which would eliminate any such difference and result in consistent models. The 3D models for all patients (ascending aorta, and whole aorta) were exported as STL files for further analysis.

### 2.3. Statistical Shape Modelling

The open-source statistical shape analysis software based on deformation vectors, ‘Deformetrica’ (Deformetrica 4, www.deformetrica.org) was used. MATLAB (Mathworks, Natick, MA, USA, MATLAB R2017a) scripts for registration and atlas construction using Deformetrica functions by Bruse et al. were used [21]. The STL files, which were then converted into VTK format, were first rigidly registered (i.e., aligned using the barycentre) for atlas construction. Atlas here refers to the template or mean shape of the population in addition to the deformations of the input 3D models. Stiffness (λ_V_) and resolution (λ_W_) parameters must then be set. Stiffness controls the elasticity of the deformations, with small λ_V_ yielding more elastic deformations [22]. Resolution controls the size of the shape features that will be included in the analysis, with small λ_W_ including finer anatomical details [22]. To calculate the optimal resolution and stiffness, the surface area of the smallest model in the population (for both the ascending aorta and whole aorta) was calculated. After calculating and setting stiffness and resolution, the atlas was constructed with a template (i.e., the mean shape of the population) and with PCA shape modes. The suitable number of modes was estimated by looking at the cumulative inertia and choosing arbitrarily a sufficient number of modes that represent a large enough percentage of shape variability (e.g., >70%). The details of the SSM methodology can be found in [21].

### 2.4. Cluster Analysis

Statistical computing software R (R 4.3, R Foundation for Statistical Computing, Vienna, Austria) was used to perform unsupervised hierarchical clustering. First, the XYZ coordinates for each shape were extracted from the SSM data and converted into vectors using the vector conversion function in R. These vectors, parametrizing each subject’s 3D shape features, constituted the input for the clustering. The clustering algorithm works by treating each object as a single node at the bottom of the dendrogram and then pairing each object using a specified linkage method at a specified distance. The pairings are repeated for (total objects in the data—1), eventually giving a single node at the top of the dendrogram, including all the objects.

The construction of the dendrogram is influenced by two parameters: the distance metric and the linkage method, which determine how close or similar the samples are to each other [23]. The distance metric relates to the distance between two points, while the linkage method defines how the objects are grouped together. The distance between two objects is relative based on the metric used; the same two objects could be close to each other using a specific distance or far away from each other using different settings, affecting the appearance and number of clusters [24]. The most commonly used distance metrics are Euclidean, Manhattan, Correlation, and Cosine, to name a few [25]. The most common linkage methods are single, complete, average, centroid, median, Ward’s, and weighted [26]. Table 1 summarises distance metrics and linkage methods. Here, we chose the correlation distance metric and the McQuitty/WPGMA linkage method because of their high accuracy and specificity, as they have been shown to be adequate for Deformetrica-generated shape parameterizations in terms of separating clinically meaningful shape clusters [12]. The correlation distance is good at assessing the similarity of shapes, which is the objective of this study. 

### 2.5. Data Analysis

Dunn’s pairwise comparison and Kruskal-Wallis test were performed to compare between the different surgical groups in regards to PCA shape modes and patients’ demographics, in addition to aortic geometric analyses between clusters. In the univariate regression, we correlated Mode 1, which represents aortic size, with patient demographics (age, BSA, BMI, height). Concerning multiple regression, we correlated Mode 1 with BSA, BMI, and height while always controlling for age.

Pearson’s chi-squared test was performed to assess the distribution frequency of surgical groups in each cluster. A *p* value of 0.05 was considered statistically significant. Statistical analyses were performed in Stata (StataMP 17, StataCorp, College Station, TX, USA).

## 3. Results

### 3.1. Patients Characteristics

The pre-operative population included 47 patients: 15 AVR, 15 Ozaki, 13 Ross, and 4 VS. The mean age was 44 years (AVR: 46 years; Ozaki: 51 years; Ross: 31 years; VS: 56 years) with a range of 16–78 years. There were 30 males and 17 females; 28 of the patients had a bicuspid aortic valve and 13 had a tricuspid aortic valve. Six did not have clear valve morphologies.

The post-operative population included 35 patients: 12 AVR, 10 Ozaki, 10 Ross, and 3 VS. The mean age was 44 years (AVR: 49 years; Ozaki: 50 years; Ross: 31 years) with a range of 17–76 years. There were 25 males and 10 females; 15 patients had a bicuspid aortic valve and 10 had a tricuspid aortic valve. Ten did not have clear valve morphology.

Patient characteristics are reported in Table 2.

### 3.2. Pre-Operative SSM Analysis

**Figure 1:**
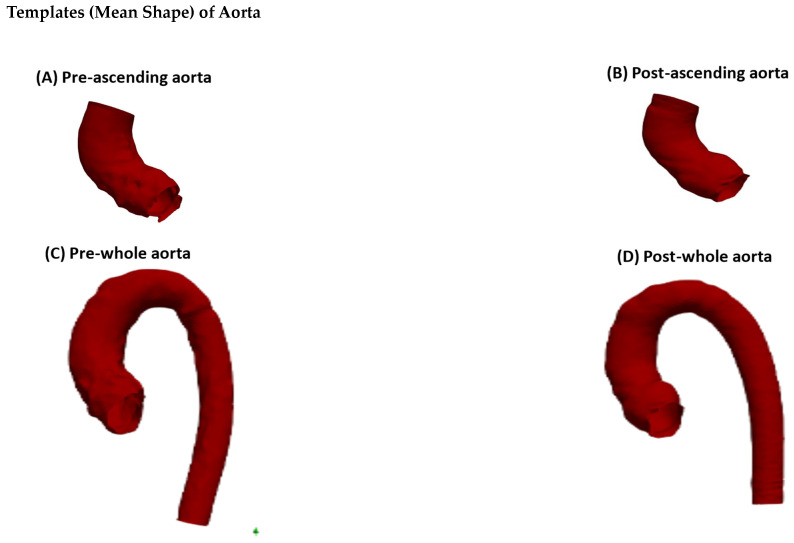
Templates of ascending and whole aortas pre- and post-operatively. (**A**) Pre-operative ascending aorta template from 47 aortas. Lambda stiffness and resolution: 35 mm and 11 mm, respectively. (**B**) Template of post-operative ascending aorta from 35 ascending aortas. Lambda stiffness and resolution: 33 mm and 11 mm, respectively. (**C**) Template of pre-operative whole aorta from 46 patients after excluding the patient with stent. Lambda stiffness and resolution: 45 mm and 11 mm, respectively. (**D**) Template of post-operative whole aorta from 35 aortas. Lambda stiffness and resolution: 45 mm and 13 mm, respectively.

The SSM produced a template of the ascending aorta from 47 ascending aortas, which depicts a relatively dilated ascending aorta with a wide sinus (Figure 1A). There were significant differences between the four surgical groups in terms of patients’ aortic size. The greatest difference was between the Ross and VS groups in terms of the PCA shape mode 1 (*p* < 0.001). The Ross included a few small-sized ascending aortas, while the VS included some of the largest ascending aortas.

Mode 1 (size) correlated with age (*p* = 0.005, r^2^ = 0.16). Using bivariate correlation analysis of age and height, the *p*-values are 0.009 and 0.087, respectively. There is a correlation with BSA (*p* = 0.02, r^2^ = 0.29) when age is included in the analysis.

Concerning the size of the whole aorta, the template produced from *n* = 46 aortas (one patient who had stented aorta that produced a low-quality 3D model was excluded) depicts a long ascending and descending aorta that is relatively thin, but with a wide aortic arch (Figure 1C). Again, there were significant differences between the size of the whole aorta in the four groups, particularly in the Ross vs. other groups (*p* < 0.001). There was no significant difference between the other groups (AVR vs. Ozaki: *p* = 0.26; AVR vs. VS: *p* = 0.19; Ozaki vs. VS: *p* = 0.14).

There was a strong correlation between Mode 1 and age (*p* < 0.001, r^2^ = 0.46). Bivariate correlation analysis with age and height has shown that height has strong correlation with the size of the whole aorta (*p* = 0.001). BSA had a strong correlation as well (*p* = 0.002), but not BMI (*p* = 0.19).

**Figure 2:**
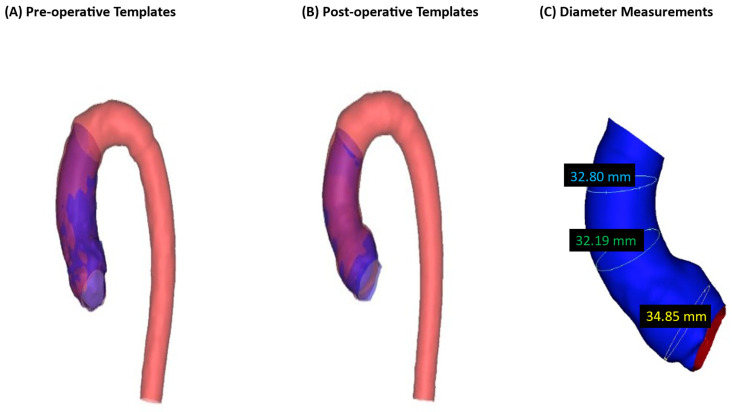
The templates of the ascending aortas and whole aortas superimposed. (**A**) Templates of the pre-operative ascending and whole aorta. (**B**) Templates of the post-operative ascending and whole aortas. (**C**) Illustrates diameter measurements’ locations: aortic root, mid and distal ascending aorta.

Qualitative (visual) similarity between ascending aorta vs. whole aorta templates is shown in Figure 2.

Three measurements were taken at the aortic root, the mid-ascending aorta, and the distal part of the ascending aorta (Figure 2C) for both the ascending aorta model and the whole aorta model in order to measure the diameter difference. This was carried out to evaluate the SSM’s template construction accuracy. For the pre-operative ascending aorta, the measurements were: 36.19 mm, 39.48 mm, 36.57 mm; for the whole aorta, the measurements were: 36.19 mm, 39.53 mm, 35.68 mm, indicating a sub-millimetre difference overall. For the post-operative ascending aorta, the measurements were: 34.85 mm, 32.19 mm, 32.80 mm; for the whole aorta, the measurements were: 33.60 mm, 33.56 mm, 33.36 mm, indicating a difference ranging from sub-millimetre to 1.37 mm. This signifies that the SSM was accurate in producing matching templates (ascending aorta and whole aorta) despite different lambda parameters for each. Aortic diameter measurements are reported in Table 3.

### 3.3. Post-Operative SSM Analysis

The post-operative SSM analysis produced a template from a population sample of 35 ascending aortas, which depicted an ascending aorta with a slightly wide sinus (Figure 1B). The differences between the surgical groups in terms of aortic size were not as great as in the pre-operative groups. The greatest difference in terms of PCA shape mode 1 is between the AVR group and the Ross group (*p* = 0.02), and between the AVR group and the Ozaki group (*p* = 0.04). Differences in aortic size between other groups were not significant (AVR vs. VS: *p* = 0.30; Ozaki vs. Ross: *p* = 0.40; Ozaki vs. VS: *p* = 0.26; Ross vs. VS: *p* = 0.20).

**Figure 3:**
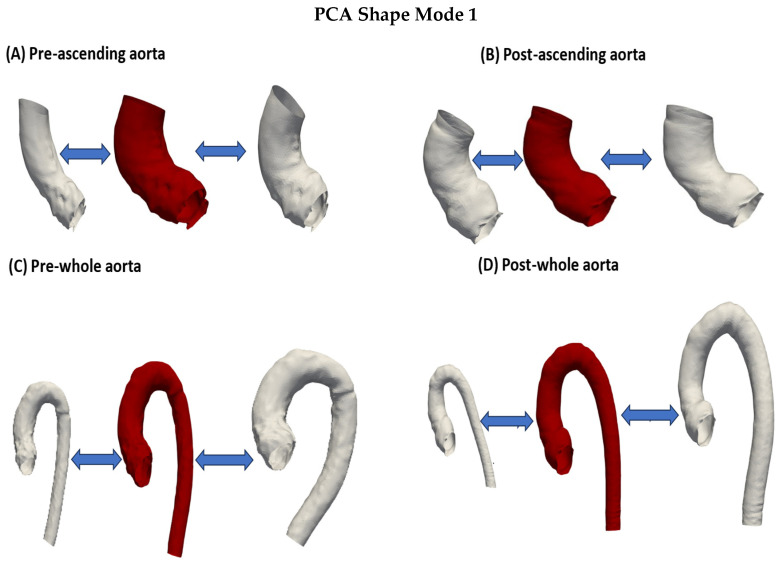
The first mode of the PCA shape modes depicting ascending and whole aortas pre- and post-operatively. The three aortas from left to right illustrate −2 standard deviation, the mean shape, and +2 standard deviation. This mode depicts mostly the size of the aorta. (**A**) Pre-operative ascending aorta. The shape deforms from a small thin ascending aorta with a relatively thin sinus to a large and dilated ascending aorta that is also curved and has a wide aortic root. (**B**) Post-operative ascending aorta. (**C**) Pre-operative whole aorta. Here, a more tortuous descending aorta is seen. The shape deforms from a thin whole aorta to a larger and more dilated whole aorta with tortuous descending aorta. (**D**) Post-operative whole aorta. Again, a slightly tortuous descending aorta is shown at +2 standard deviation.

As opposed to the pre-operative results, the correlation of aortic size, represented in mode 1, with age, was not significant (*p* = 0.07). In the univariate correlation, height was the only factor that had a significant correlation with mode1/aortic size (*p* = 0.01). Bivariate correlation analysis was also not significant except for age when also correlated with BMI (*p* = 0.05), and height when also correlated with age (*p* = 0.02). Mode 1 is illustrated in Figure 3. Modes 2–4 are shown in the Appendix A.

For the whole aorta, the SSM analysis produced a template that depicted an aorta that had a relatively long ascending aorta with a slightly wide sinus and a descending aorta that was relatively thin (Figure 1D). Similar to the pre-operative aortas, the smallest aorta belonged to the Ross group, while the largest belonged to the VS group. In PCA shape mode 1, the Ross group showed the strongest difference from all other groups (vs. AVR: *p* = 0.04; vs. Ozaki: *p* = 0.02; vs. VS: *p* = 0.03). Apart from this, there was no statistical significance between the groups in terms of aortic size (AVR vs. Ozaki: *p* = 0.32; AVR vs. VS: *p* = 0.24; Ozaki vs. VS: *p* = 0.34).

Univariate correlation analysis showed that age strongly correlated with the size of the whole aorta (*p* < 0.001, r^2^ = 0.47). Both BSA and height also correlated with size (*p* = 0.03 and *p* = 0.009, respectively). However, BMI had no correlation with size (*p* = 0.80).

**Figure 4:**
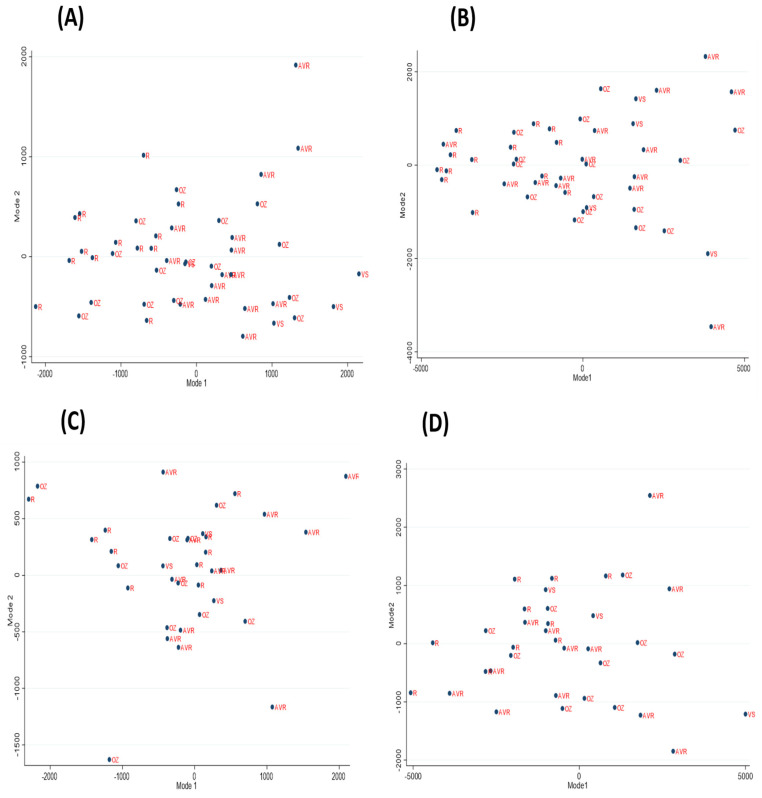
PCA scatterplots of Mode 1 vs. Mode 2 for the pre-operative and post-operative ascending and whole aortas. (**A**) Pre-operative ascending aortas; (**B**) Pre-operative whole aortas; (**C**) Post-operative ascending aortas; (**D**) Post-operative whole aortas. In all scenarios, there is no clear distinction between the surgical groups, except for the Ross where they tend to mostly cluster together. The Ross shapes have a tendency of having negative values for Mode 1 suggesting small aortas. This clustering also shows that the Ross have less variability compared to the other surgical groups. Many of the AVR points also seem to cluster together, but other points are also dispersed throughout the plot, indicating greater variability. The Ozaki points are moderately spread out but less in comparison to the AVR group. The valve-sparing group is too small to yield meaningful results. AVR: aortic valve replacement; OZ: Ozaki; R: Ross; VS: valve-sparing.

A PCA scatterplot of Mode 1 vs. Mode 2 for the pre-operative and post-operative ascending and whole aortas was carried out to identify shape variability between the surgical groups (Figure 4).

Table 4 describes in detail the cumulative inertia and PCA shape mode for the pre-operative and post-operative analyses.

### 3.4. Pre-Operative Cluster Analysis

**Figure 5:**
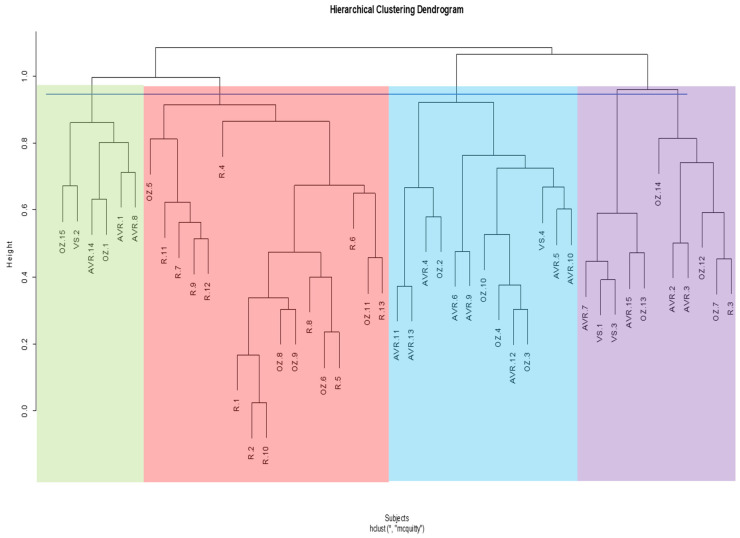
Hierarchical cluster dendrogram of pre-operative ascending aortas. Height represents extent of dissimilarity between clusters. Cut-off line was placed at height of ~1.0, resulting in four clusters. Linkage method and distance metric used were McQuitty (WPGMA) and correlation respectively. AVR: aortic valve replacement; OZ: Ozaki; R: Ross; VS: valve-sparing. The numbers following acronyms represent patient number. * Hierarchical cluster analysis; McQuitty linkage method and correlation distance.

For the pre-operative cluster analysis ascending aorta, the dendrogram divides into two main clusters, which further subdivides into five subclusters (Figure 5). Using Pearson’s chi-squared test to assess the frequency of distribution, we show that the difference between the five clusters in terms of encompassing the proportion of aortas belonging to the four surgical groups is statistically significant (*p* < 0.001).

**Figure 6:**
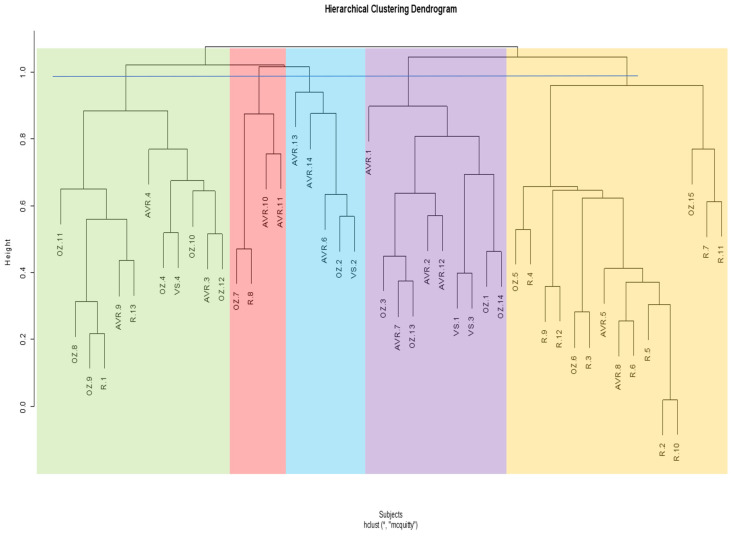
Hierarchical cluster dendrogram of pre-operative whole aortas. Height represents extent of dissimilarity between clusters. Cut-off line was placed at a height of ~1.0, resulting in five clusters. Linkage method and distance metric used were McQuitty (WPGMA) and correlation, respectively. AVR: aortic valve replacement; OZ: Ozaki; R: Ross; VS: valve-sparing. The numbers following acronyms represent patient number. * Hierarchical cluster analysis; McQuitty linkage method and correlation distance.

In the pre-operative cluster analysis whole aorta, the dendrogram divides into two primary clusters, which are then subdivided into five subclusters (Figure 6). Using Pearson’s chi-squared test to assess the frequency of distribution, we show that the difference between the five clusters in terms of encompassing the proportion of aortas belonging to the four surgical groups is statistically significant (*p* = 0.03). A contingency table of the pre-operative ascending and whole aortas within clusters is shown in Table 5.

**Figure 7:**
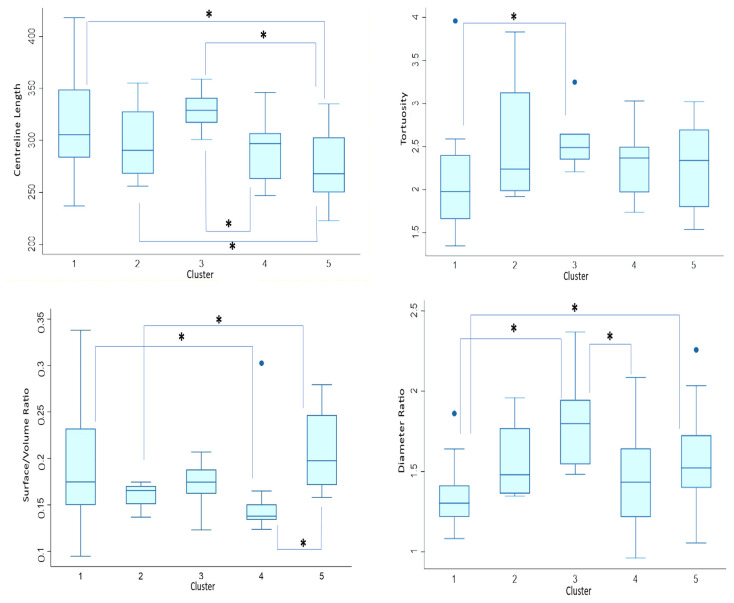
Pre-operative Dunn’s Pairwise comparison represented by boxplots of centreline length, tortuosity, surface area/volume ratio, and ascending/descending aorta diameter ratio by cluster. Median value is represented by line within box, height of box demonstrates interquartile range, and whiskers demonstrate maximum and minimum values. * signifies statistical significance of *p* < 0.05. Blue dots signify outliers.

We wanted to assess how distinct the clusters are from each other, so we decided to test for geometric variations of the whole aortas within the clusters. Cluster III is significantly different from clusters IV and V (*p* = 0.05; *p* = 0.004), which is in turn different from clusters I and II (*p* = 0.02) in terms of centreline length. Concerning tortuosity, the only significant difference is between clusters I and III (*p* = 0.02). In terms of surface area/volume ratio, there is a significant difference between cluster IV and clusters I and V (*p* = 0.02; *p* < 0.001), and cluster II is also significantly different from cluster V (*p* = 0.05). Regarding ascending/descending aorta diameter, there is a significant difference between cluster I and clusters III and V (*p* = 0.002; *p* = 0.01), and between cluster III and cluster IV (*p* = 0.02). Figure 7 illustrates this in detail.

### 3.5. Post-Operative Cluster Analysis

**Figure 8:**
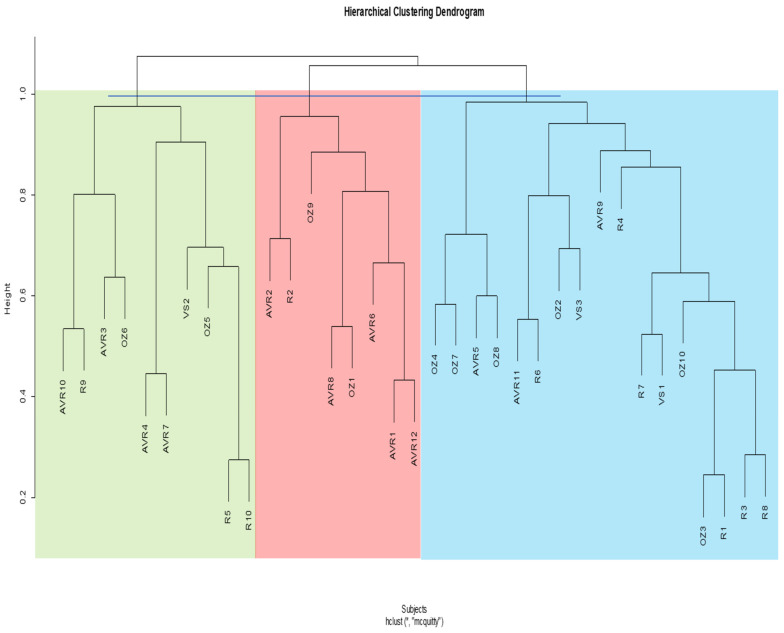
Hierarchical cluster dendrogram of post-operative ascending aortas. Height represents extent of dissimilarity between clusters. Cut-off line was placed at a height of ~1.0 resulting in five clusters. Linkage method and distance metric used were McQuitty (WPGMA) and correlation, respectively. AVR: aortic valve replacement; OZ: Ozaki; R: Ross; VS: valve-sparing. The numbers following acronyms represent patient number. * Hierarchical cluster analysis; McQuitty linkage method and correlation distance.

In the post-operative cluster analysis ascending aorta, the dendrogram is divided into two main clusters, which are then subdivided into five subclusters (Figure 8). This time, the Pearson’s chi-squared test does not show a significant difference between the clusters (*p* = 0.47).

**Figure 9:**
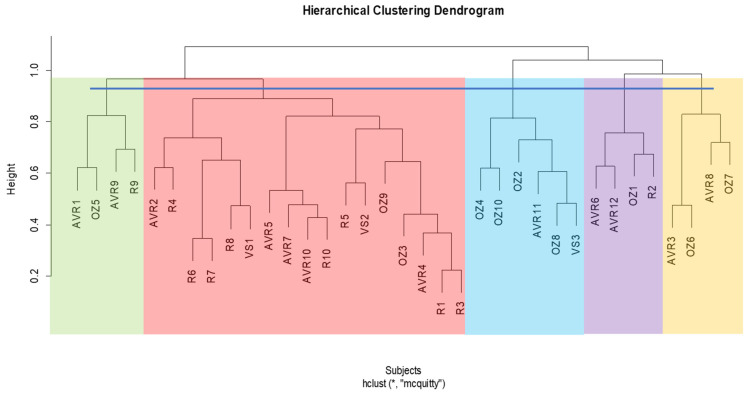
Hierarchical cluster dendrogram of post-operative whole aortas. Height represents extent of dissimilarity between clusters. Cut-off line was placed at a height of ~0.95–1.0 resulting in five clusters. Linkage method and distance metric used were McQuitty (WPGMA) and correlation respectively. AVR: aortic valve replacement; OZ: Ozaki; R: Ross; VS: valve-sparing. The numbers following acronyms represent patient number. * Hierarchical cluster analysis; McQuitty linkage method and correlation distance.

For the post-operative cluster analysis whole aorta, the dendrogram is divided into two primary clusters, which is then subdivided into five subclusters (Figure 9). Again, there is no significant difference between the clusters (*p* = 0.19). A contingency table of post-operative ascending and whole aortas within clusters is shown in Table 6.

**Figure 10:**
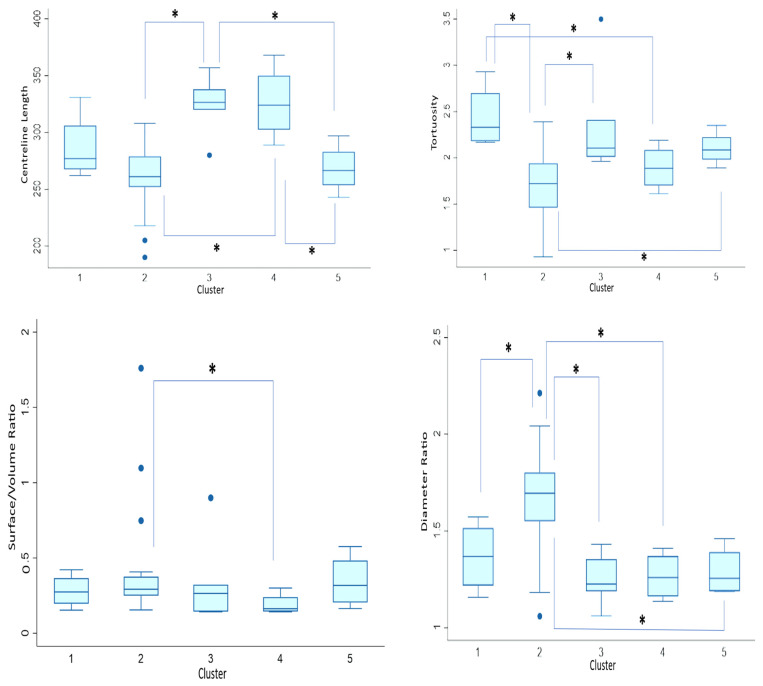
Post-operative Dunn’s Pairwise comparison represented by boxplots of centreline length, tortuosity, surface area/volume ratio, and ascending/descending aorta diameter ratio by cluster. Median value is represented by line within box, height of box demonstrates interquartile range, and whiskers demonstrate maximum and minimum values. * signifies statistical significance of *p* < 0.05. Blue dots signify outliers.

Concerning geometric analyses within clusters, Figure 10 demonstrates a significant difference between cluster II and clusters III and IV in terms of centreline length (*p* < 0.001). There is also a significant difference between cluster V and both clusters III and IV (*p* = 0.02). Concerning tortuosity, there is a significant difference between cluster I and both clusters II and IV (*p* < 0.001; *p* = 0.03). Cluster II is significantly different between cluster III and V (*p* = 0.002; *p* = 0.03). There is also a significant difference between cluster II and cluster IV (*p* = 0.02) in terms of surface area/volume ratio. Regarding ascending/descending aorta diameter, the second cluster stands out as it is significantly different from all the other clusters (*p* = 0.05; *p* = 0.003; *p* = 0.008; *p* = 0.02). Given the above information, it seems that cluster two is the most distinct from the other clusters. Interestingly, almost half of the shapes within this cluster are Ross, which may indicate that Ross patients have markedly different aortic morphology.

## 4. Discussion

This study presented the feasibility of applying both 3D statistical shape modelling and unsupervised hierarchical clustering to a population of patients before and after AVR surgery, assessing if such methods could be useful in detecting differences in the aortic morphology due to different surgical procedures. For both methods, we assessed the ascending aorta as well as the whole aorta.

In the SSM, we produced a template of the ascending aorta and the whole aorta. Next, we calculated the shape modes based on PCA. Compared to pre-operative ascending aortas, there is less variability between the ascending aortas post-operatively. However, the different surgical procedures seem to influence different aortic deformations. For example, in the first mode, the AVR procedure group has the largest aortic size, while the smallest ascending aorta belongs to the Ross group. We wanted to make sure if the size of the ascending aorta is related to the procedure itself or if it is simply due to the patients’ demographics and anthropometrics. Even though there was a significant age difference specifically between the Ross group, which includes the subject with the smallest aortic surface area, and the AVR and Ozaki groups, we found no correlation between age and aorta size (*p* = 0.07). We also found no correlation with BMI or BSA. However, there was a correlation with height (*p* = 0.01). This shows that the size of the ascending aorta is mostly attributed to the patient’s height and is not associated with the surgical procedure.

Concerning the whole aorta, we saw greater shape variability, even in the first mode, which usually shows only changes in length and dilatation, but here not only does the whole aorta become larger in size, but the tortuosity of the descending aorta also increases, resulting in a ‘question mark’ appearance. We found that size here is correlated with age and BSA as well as height, perhaps due to the inclusion of a larger portion of the aorta. Again, the smallest whole aorta is influenced by the Ross group, but the largest aorta is influenced by the VS group in this case. This is different from the ascending aorta results because perhaps the aortic arch and descending aorta are larger in the VS compared to the AVR.

Hierarchical clustering is not the only method that could be used to classify objects, e.g., K-means clustering. We chose to use hierarchical clustering as opposed to K-means clustering since K-means clustering requires specifying the number of clusters, while hierarchical clustering does not have such a prerequisite. In addition, K-means clustering is sensitive to noise which could affect the quality of clustering [27]. We wanted the method to be completely unsupervised, which is especially helpful when we know little about the interdifferences and variations within the population under study. Another advantage of hierarchical clustering is that one can identify subclusters within the clusters due to its hierarchical representation [28].

Our choice of settings was based on studies conducted by Bruse et al. [12] and Gundelwein et al. [29] as Bruse and colleagues have shown that the use of correlation distance and the weighted method was found to have the highest specificity and accuracy above 90% [12]. The McQuitty method was also shown to have the best performance compared to other methods in one study [30]. We chose the correlation distance because Lance and Williams [31] recommended that the use of the correlation distance is quite useful when the researcher wants to assess the interrelationship between objects, such as the shapes of the objects, which is the purpose of our clustering. Additionally, two previous studies that also assessed aortas used the correlation distance and were successful in classifying the aortas according to their pathology [12,29].

The algorithm of the clustering analysis automatically classifies the shapes into a number of clusters based on their morphology; the number need not be specified, which is why it is considered unsupervised; however, we have to specify where to cut off the tree, which affects the interpretability of the results, since the number of clusters will change. That is, the higher the cut-off line on the dendrogram, the fewer the clusters where all shapes are clustered in one group, and the lower the cut-off line, the more clusters since it is closer to individual shapes. We tried to choose the maximum height possible, indicating the greatest difference between the clusters, but below the height where the tree splits into two clusters since comparing two clusters is not informative.

For the pre-operative ascending aorta, we found four clusters, while we found five clusters for the whole aorta. This suggests a great difference between the ascending and whole aortas, even though they belong to the same patients. Similar to the construction of the SSM atlas, the clustering will be influenced by the degree of morphological variations, and perhaps the difference between the dendrograms implies that there is a greater difference between the patients within the region of the ascending aorta than the region of the aortic arch and descending aorta. Since the ascending aorta is smaller, the clustering analysis will be more sensitive to subtle variations within that region that may be caused by valve pathology, and the inclusion of the arch and descending aorta may decrease the sensitivity.

For the post-operative ascending and whole aorta, we attained three and five clusters, respectively. The dendrograms did not cluster the shapes based on the different surgical procedures, except for most of the Ross shapes. Six out of ten Ross ascending aortas were clustered in one group, while eight out of ten were clustered in one group for the whole aorta. Interestingly, this kind of clustering also occurs in the pre-operative analysis, where 12 out of 13 Ross ascending aortas cluster in one group and 10 out of 13 cluster for the whole aorta. This shows that the hierarchical clustering methodology can successfully classify aortas based on morphological variance since we already know that (1) there is no big difference between the AVR, Ozaki, and VS procedures in terms of significant morphological changes that they may cause, and (2) that the Ross procedure is quite significantly different from the other procedures. However, since the pre-Ross shapes do also cluster together and are also considered different from the other procedures, this may indicate that it may not necessarily be due to the surgery itself.

Additionally, we assessed geometric differences between the clusters in terms of centreline length, tortuosity, surface area/volume ratio, and ascending/descending aorta diameter ratio. Indeed, there were significant morphological differences between the found shape clusters, as shown by analysing the respective morphometric parameters.

### 4.1. Limitations

This was a feasibility study, and the sample size was too small to produce clinically significant results. Therefore, clinical outcomes were not assessed. We had two different patient cohorts for the pre-operative and post-operative analyses. If data were available pre- and post-surgery for the same patient (or, in our case, across the two cohorts) it would be possible to compare how the aortas change structure due to the surgical procedure using a longitudinal SSM approach [32]. This is due to a lack of imaging data, as scans were routine clinical scans, not research scans.

### 4.2. Future Outlook

We have shown that SSM and hierarchical clustering could be used to assess and classify aortic morphology pre- and post-operation. The classification could help clinicians in patient risk stratification, leading to better diagnosis, and treatment plans. The assessment of post-operative structures could also show how certain surgical procedures change the anatomy, which can impact cardiovascular function. In this way, researchers and surgeons could choose the most appropriate surgical procedure, modify it, or even come up with a novel, more optimised procedure. Researchers could design customised valve prostheses that closely match the patients’ anatomy. These statistical methods could be applied to any cardiovascular structure and any surgical treatment.

## 5. Conclusions

We demonstrated the feasibility of applying statistical shape modelling and hierarchical clustering to evaluate aortic morphology before and after different aortic valve surgeries, namely the traditional AVR, the Ozaki, Ross, and valve-sparing procedures. We have shown that the Ross surgical group has aortic variability that is different compared to the rest. Clustering has shown that this difference exists in both pre- and post-operation, potentially indicating the aortic variability is not solely due to the surgical procedure itself but to this specific cohort of patients who were chosen to undergo the Ross procedure. In the future, researchers could progress on this work by including large sample sizes to yield clinically meaningful results that may help surgeons and clinicians improve surgical treatment and patients’ quality of life.

## Figures and Tables

**Table 1:** A summary of the common linkage method and distance metrics used in hierarchical cluster analysis. The *x* and *y* in the equations represent coordinates of points in a 2D space.

Linkage Method	Description	Distance Metric	Description	Distance Equation
Single	The objects are grouped in one cluster depending on the two closest objects within the clusters. Produces long clusters.	Euclidean	Based on the Pythagoras theorem formula and is used when data are continuous and have normal distribution. High values will be clustered together, and low values will be clustered together.	$d\left(x,y\right)=\sum {\left(x_{i}-y_{i}\right)}^{2}$
Complete	The opposite of single; the objects are grouped based on the furthest objects within the clusters. Produces compact spherical clusters.	Manhattan	It measures by summing the absolute differences of the coordinates of two objects.	$D_{man}=\sum_{i=1}^{n}(x_{i}-y_{i})$
Average	This is based on the average distance of the objects within the clusters.	Correlation	Based on the correlation coefficient. Most common is the Pearson parametric correlation. Sensitive to outliers.	$\begin{array}{l} d_{correlation}\\ =1-\frac{\left(x_{i}-{\bar{x}}_{i})(y_{i}-\bar{y_{i}}\right)}{\surd (x_{i}-{\bar{x}}_{i}){\left(x_{i}-{\bar{x}}_{i}\right)}^{\prime }\surd (y_{i}-{\bar{y}}_{i})(y_{i}-{\bar{y}}_{i})} \end{array}$
Centroid	The objects are grouped in a cluster based on the distance between the objects that are in the centre of the cluster.	Mahalanobois	Takes normalisation of data into account and is based on t-score.	$d_{Mahalanobois}=\surd {\left(x-\mu \right)}^{T}S^{-1}(x-\mu )$ where *S* is the covariance matrix of the distribution, µ is the mean of vector of the distribution, and ${\left(x-\mu \right)}^{T}$ is the transpose of the difference vector.
Ward	The objects are grouped based on the minimal increase in sum-of squares. Minimises the variance within each cluster.	Cosine	Measures cosine angle between two vectors.	$d_{cosine}=\frac{A\cdot B}{\left\|A\right\|\cdot \left\|B\right\|}$ where *A* and *B* are vectors and $\left\|A\right\|\cdot \left\|B\right\|$ are the magnitudes of the vectors (sum of squares).
McQuitty/Weighted (WPGMA)	Based on the average distance between clusters but does not take number of points in the clusters into consideration.			

**Table 2:** Shows pre-operative and post-operative patients’ demographics and anthropometrics, including valve morphology. Values are mean ± SD. BAV: bicuspid aortic valve; TAV: tricuspid aortic valve. AVR: aortic valve replacement (traditional); VS: valve-sparing.

Pre-Operative Patient Characteristics				
Variable	AVR (*n* = 15)	Ozaki (*n* = 15)	Ross (*n* = 13)	VS (*n* = 4)
Sex	12 m; 3 f	12 m; 3 f	3 m; 10 f	3 m; 1 f
Age (years; mean ± SD)	46.0 ± 19.0	51.0 ± 12.0	31.0 ± 11.0	56.0 ± 7.0
Height (cm)	174.7 ± 6.4	176.3 ± 10.9	167.0 ± 9.5	174.3 ± 9.0
Weight (kg)	81.8 ± 16.6	88.0 ± 18.8	73.1 ± 15.9	78.3 ± 19.9
BSA (m^2^)	2.0 ± 0.2	2.0 ± 0.2	1.9 ± 0.2	1.9 ± 0.3
BMI (kg/m^2^)	26.9 ± 5.4	28.0 ± 5.6	26.2 ± 5.8	25.5 ± 3.9
Valve Type	8 BAV; 6 TAV	10 BAV; 3 TAV	10 BAV; 2 TAV	2 TAV
Ascending Aorta Surface Area (mm^2^)	10,989.0 ± 2711.0	13,421.0 ± 2726.0	9031.0 ± 2125.0	14,658.0 ± 3125.0
Whole Aorta Surface Area (mm^2^)	32,570.0 ± 7880.0	29,969.0 ± 6292.0	22,221.0 ± 4180.0	34,841.0 ± 6151.0
**Post-Operative Patient Characteristics**				
**Variable**	**AVR (*n* = 12)**	**Ozaki (*n* = 10)**	**Ross (*n* = 10)**	**VS (*n* = 3)**
Sex (M, F)	12 m; 0 f	6 m; 4 f	5 m; 5 f	2 m; 1 f
Age (years, mean ± SD)	49.5 ± 17.2	49.9 ± 10.8	31 ± 14.2	47.3 ± 27.3
Height (cm)	177.8 ± 4.7	173.4 ± 13.2	163.6 ± 10.1	184 ± 7.1
Weight (kg)	85.2 ± 16.7	85.8 ± 21.4	76.3 ± 15.9	75.5 ± 16.3
BSA (m^2^)	2.0 ± 0.2	2.0 ± 0.3	1.8 ± 0.2	2.0 ± 0.2
BMI (kg/m^2^)	26.8 ± 4.7	28.6 ± 7.1	29.2 ± 9.0	22.6 ± 6.6
Valve Type	5 BAV; 2 TAV	7 BAV; 2 TAV	3 BAV; 4 TAV	2 TAV
Ascending Aorta Surface Area (mm^2^)	10,794.0 ± 1977.0	9695.0 ± 1640.0	8029.0 ± 2118.0	9212.0 ± 1761.0
Whole Aorta Surface Area (mm^2^)	27,336.0 ± 6239.0	27,011.0 ± 4747.0	20,409.0 ± 4460.0	30,233.0 ± 11,028.0

**Table 3:** The diameter measurements of the pre-operative and post-operative templates of the ascending aortas and whole aortas.

Templates	Aortic Root Diameter	Mid-Ascending Aorta Diameter	Distal Ascending Aorta Diameter
Pre-operative Ascending Aorta	36.19 mm	39.48 mm	36.57 mm
Pre-operative Whole Aorta	36.19 mm	39.53 mm	35.68 mm
Post-operative Ascending Aorta	34.85 mm	32.19 mm	32.80 mm
Post-operative Whole Aorta	33.60 mm	33.56 mm	33.36 mm

**Table 4:** Cumulative Inertia and PCA Shape Modes. Displays the cumulative inertia and PCA shapes of ascending and whole aortas in pre- and post-operative analyses.

Pre-Operative Analysis				
Scenario	Mode 1	Mode 2	Mode 3	Mode 4
Cumulative Inertia—Ascending Aorta (Contribution)	24% (24%)	36% (12%)	47% (11%)	57% (10%)
Cumulative Inertia—Whole Aorta (Contribution)	27% (27%)	38% (11%)	47% (9%)	55% (8%)
Ascending Aorta Shape features	Small and thin > large and dilated	Wide aortic root > elongated segment proximal to aortic arch	Thin and long section proximal to aortic arch > wide and short aorta and narrow sinus	Narrow aortic root and long aorta > wide aortic root and short aorta
Whole Aorta Shape	Small > large and dilated, curved descending aorta	‘Hook-like’ appearance > tortuous + dilated descending aorta	Dilated ascending > thin ascending aorta	Long and narrow aorta, gothic arch > short and dilated aorta, crenel arch
**Post-Operative Analysis**				
**Scenario**	**Mode 1**	**Mode 2**	**Mode 3**	**Mode 4**
Cumulative Inertia—Ascending Aorta (Contribution)	24% (24%)	36.3% (12.3%)	46% (9.7%)	54.6% (8.6%)
Cumulative Inertia—Whole Aorta (Contribution)	25.6% (25.6%)	36.6% (11%)	46.6% (10%)	54.6% (8%)
Ascending Aorta Shape	Small > large	Curved, slightly long > less curvature, short, dilated	Curved and dilated > slightly narrower	Dilated and curved > thinner
Whole Aorta Shape	Small > large, tortuous descending aorta	Dilated and long ascending, short descending, tortuous > short ascending, long descending aorta	Long ascending, short and wide descending > short ascending, long descending	Curved aorta, wide aortic root > long and straight descending, thin aortic root, wide ascending aorta

**Table 5:** Pre-operative Contingency Table of surgical groups within clusters.

Ascending Aortas						
Group	Cluster I	Cluster II	Cluster III	Cluster IV		Total
AVR	3	0	8	4		15
Ozaki	2	5	4	4		15
Ross	0	12	0	1		13
VS	1	0	1	2		4
Total	6	17	13	11		47
**Whole Aortas**						
**Group**	**Cluster I**	**Cluster II**	**Cluster III**	**Cluster IV**	**Cluster V**	
AVR	3	2	3	4	2	14
Ozaki	6	1	1	4	3	15
Ross	2	1	0	0	10	13
VS	1	0	1	2	0	4
Total	12	4	5	10	15	46

Contingency table of surgical groups within clusters. Pearson’s chi-squared test. X^2^ (9) = 30.88 indicating *p*-value of <0.01 for ascending aortas. X^2^ (12) = 22.64 indicating *p*-value of 0.03 for whole aortas. Numbers in the table indicate the number of shapes corresponding to each surgical procedure within each cluster.

**Table 6:** Post-operative Contingency table of surgical groups within clusters.

Ascending Aortas						
Group	Cluster I	Cluster II		Cluster III		Total
AVR	4	5		3		12
Ozaki	2	2		6		10
Ross	3	1		6		10
VS	1	0		2		3
Total	10	8		17		35
**Whole Aortas**						
**Group**	**Cluster I**	**Cluster II**	**Cluster III**	**Cluster IV**	**Cluster V**	**Total**
AVR	2	5	1	2	2	12
Ozaki	1	2	4	1	2	10
Ross	1	8	0	1	0	10
VS	0	2	1	0	0	3
Total	4	17	6	4	4	35

Contingency table of surgical groups within clusters. Pearson’s chi-squared test. X^2^ (6) = 5.80 indicating *p*-value of 0.45 for ascending aortas. X^2^ (12) = 13.55 indicating *p*-value of 0.33 for whole aortas. Numbers in the table indicate the number of shapes corresponding to each surgical procedure within the clusters.

**Figure A1:**
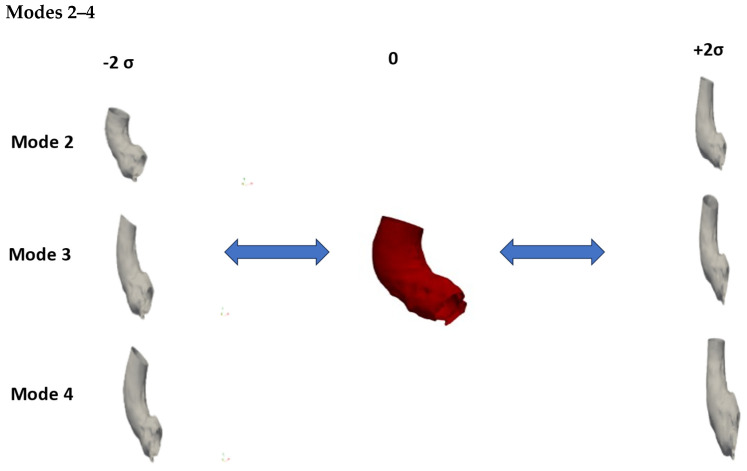
Figure shows shape modes 2–4 accounting for 33% shape variability. Aortas on the left are −2 standard deviation and aortas on the right are +2 standard deviation. The aorta in the middle is the mean shape.

**Figure A2:**
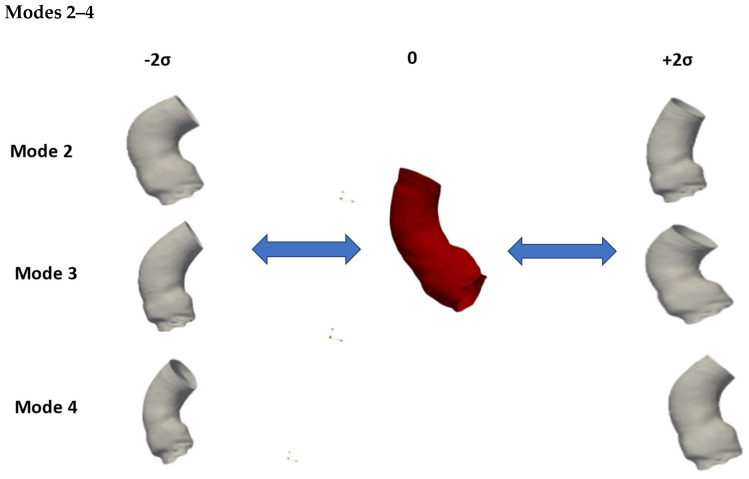
Shape modes 2–4 of post-operative ascending aortas accounting for 30.6% shape variability. Aortas on the left are −2 standard deviation and aortas on the right are +2 standard deviation. The aorta in the middle is the mean shape.

**Figure A3:**
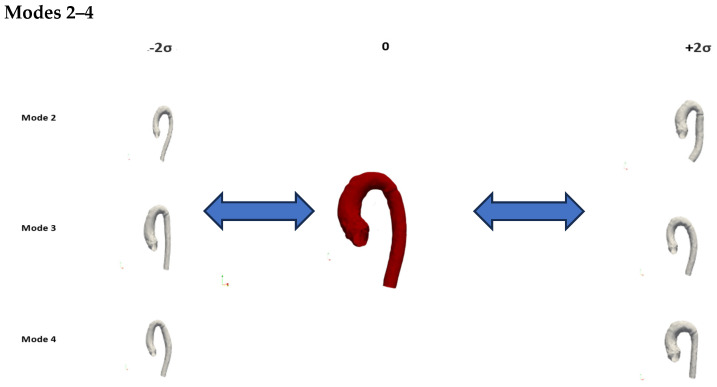
Figure shows shape modes 2–4 accounting for 28% shape variability. Aortas on the left are −2 standard deviation and aortas on the right are +2 standard deviation. The aorta in the middle is the mean shape.

**Figure A4:**
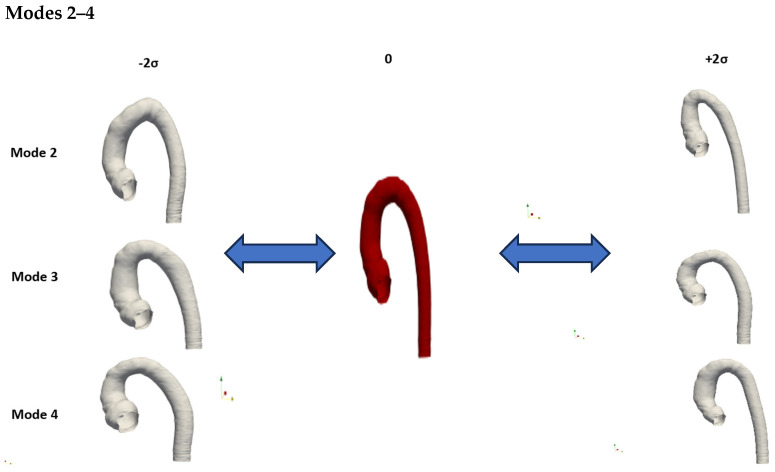
Shape modes 2–4 of the post-operative whole aortas accounting for 28% shape variability. Aortas on the left are −2 standard deviation and aortas on the right are +2 standard deviation. The aorta in the middle is the mean shape.

## Data Availability

Data are contained within the article.
